# Participation pathways in global vaccine partnerships and their implications for immunization systems: a comparative analysis of China and India in Gavi

**DOI:** 10.3389/fpubh.2026.1826364

**Published:** 2026-05-28

**Authors:** Mengli Ding

**Affiliations:** School of Politics and Public Administration, Soochow University, Suzhou, China

**Keywords:** China, emerging economies, GAVI, health systems strengthening, immunization systems, India, public health policy, vaccine access

## Abstract

**Background/objectives:**

As an increasing number of emerging economies move beyond a simple recipient role in global vaccine partnerships, their engagement with Gavi has become more diverse and institutionally layered. This study examined how different participation pathways in Gavi were associated with domestic immunization strengthening and roles in global vaccine procurement, using China and India as two comparable emerging economies with contrasting modes of engagement.

**Methods:**

A long-term qualitative comparative case study was conducted for 2000–2025 using a most-similar systems design. The analysis combined process tracing and structured cross-case comparison. Evidence was triangulated from institutional documents and public databases, Gavi and WHO materials, corporate disclosures and partnership records, peer-reviewed studies, and semi-structured interviews conducted between 2019 and 2025. The materials were manually coded around role evolution, participation mechanisms, pathway orientation, and outcome mapping.

**Results:**

Two distinct participation pathways were identified. China followed a state-coordinated pathway in which Gavi-related engagement was linked more closely to domestic system development, policy alignment, and selective donor-oriented repositioning. Corporate participation was limited and episodic, with the most visible expansion occurring during the COVID-19 period under government coordination. India followed a market-integrated pathway, characterized by sustained manufacturer participation in UNICEF–Gavi pooled procurement. This pathway was supported by WHO-prequalified products, technology-transfer arrangements, large-scale production, and cost competitiveness. The comparison suggests that China’s engagement was more closely associated with domestic system consolidation, whereas India’s engagement was associated with a stronger position in global vaccine supply but coexisted with more uneven domestic immunization-system outcomes.

**Conclusion:**

Emerging economies do not engage with Gavi through a single pathway, and these differences have important public health implications. For major Southern vaccine-producing countries, public health policy may need to better sequence global engagement with domestic system strengthening: first improving delivery, cold-chain, workforce, surveillance, and information systems; then upgrading regulatory capacity and WHO prequalification readiness; and finally linking industrial upgrading and procurement participation to equitable vaccine access.

## 1 Introduction

Global access to immunization has evolved from a predominantly intergovernmental aid model into a more complex governance architecture in which transnational public-private partnerships play a central role (1). These partnerships represent institutionalized, cross-border arrangements that convene governments, international organizations, philanthropic foundations, and pharmaceutical manufacturers to mobilize financing, coordinate markets, and position vaccines as global public goods (2). This shift is not only institutional but also operational: vaccine access now depends on the interaction among public financing, market-shaping instruments, regulatory standards, manufacturing capacity, and country-level delivery systems. Gavi, the Vaccine Alliance, is one of the most important platforms in this architecture. Since its establishment in 2000, Gavi has supported the immunization of more than 1.1 billion children and helped avert more than 18.8 million future deaths (3).

Gavi’s role is distinctive because it does not operate only as a financing mechanism. It also functions as a market-shaping and coordination platform. Through pooled procurement, multi-year demand commitments, co-financing requirements, and instruments such as Advance Market Commitments, Gavi works with the United Nations Children’s Fund (UNICEF), the World Health Organization (WHO), the World Bank, donors, recipient governments, civil society organizations, and vaccine manufacturers to reduce demand uncertainty, negotiate more affordable prices, and secure more predictable supply for low- and lower-middle-income countries (4, 5). UNICEF, as Gavi’s main procurement partner, describes its role as supporting vaccine market dynamics to ensure reliable supply of quality and affordable vaccines (6). This public-private architecture makes Gavi an especially useful case for examining how global vaccine partnerships connect aid, markets, regulation, and national immunization systems.

A further change in this system is that emerging economies are no longer linked to Gavi only as vaccine aid recipients. As their fiscal resources, manufacturing capacity, and regulatory systems develop, some countries occupy multiple roles at once: program beneficiary, self-financing country, supplier, donor, or strategic partner. India illustrates this role complexity particularly clearly. It has been one of Gavi’s largest recipient countries, while its domestic manufacturers have simultaneously emerged as central suppliers in global vaccine markets. The WHO Global Vaccine Market Report 2024 shows that the 2023 global vaccine market covered 88 vaccine products sold in 207 countries through multiple procurement channels by 116 manufacturers, and that the Serum Institute of India remained one of the dominant suppliers by volume (7). China, by contrast, moved from being a former Gavi-supported country to a financial contributor and COVID-19 Vaccines Global Access (COVAX) participant, including a US$100 million contribution to COVAX in 2022 (8). These role transitions raise a policy-relevant question: as emerging economies undergo role transitions within Gavi, what participation pathways do they follow, and how are these pathways associated with domestic immunization strengthening and with policy-relevant outcomes in global vaccine procurement systems?

China and India provide two particularly informative cases. Both are major emerging economies with long-standing engagement in Gavi and rising health and production capacity, yet they have followed markedly different participation pathways. Their comparison is analytically useful because the two countries share several broad structural similarities, but differ in how state institutions, vaccine manufacturers, regulatory entry, and procurement channels are connected to Gavi-related participation. Comparing these two cases within a most-similar systems design (9) helps identify how different forms of engagement within the same partnership framework may be associated with different public health consequences.

Existing scholarship has examined the growing role of emerging economies in global health governance—from earlier work on BRICS influence and firms from emerging markets to more recent studies on China’s, India’s, and Russia’s health or vaccine diplomacy during the COVID-19 pandemic (10–15). Much of this literature, however, remains concentrated on the pandemic period or on macro-level diplomatic narratives. Less attention has been paid to the longer-term institutional pathways through which emerging economies interact with the same global health partnership over time, and to how these pathways shape both domestic public health systems and global procurement structures. This gap matters because the consequences of partnership participation are not uniform: the same institutional platform may strengthen domestic system absorption in one country while deepening global supply-market embeddedness in another.

To address this gap, this study compares China and India within Gavi and argues that emerging economies participate through differentiated institutional pathways. China represents a more state-coordinated pathway, in which partnership engagement was primarily organized through public authorities and was more closely linked to domestic immunization system development, policy alignment, and public-sector coordination. India represents a more market-integrated pathway, in which domestic manufacturers became recurrent participants in UNICEF–Gavi pooled procurement through WHO prequalification, supply agreements, production scale, and cost competitiveness. This pathway was associated with India’s stronger role in global vaccine supply, but it coexisted with uneven domestic immunization-system consolidation, especially in delivery capacity, financing, cold-chain and logistics support, information systems, workforce capacity, and subnational implementation. By moving beyond pandemic-centered and macro-level accounts, the study provides a more fine-grained understanding of how major Southern vaccine-producing countries engage with global vaccine partnerships and what these choices may mean for vaccine access strategies, immunization system development, and public health policy.

## 2 Materials and methods

### 2.1 Research design

This study uses a qualitative comparative case study design to examine how China and India engaged with Gavi through different participation pathways, and how these pathways were associated with domestic immunization strengthening and participation in global vaccine procurement. The two cases were selected using a most-similar systems logic. This design does not assume that China and India are structurally identical. Rather, it uses their similarities on several scope conditions—large emerging-economy status, location in the Global South, vaccine-production capacity, long-standing links with Gavi, and expanding health and regulatory capacities—to make their divergent modes of engagement analytically meaningful. At the same time, major structural differences between the two countries, including state–market relations, public-sector coordination, industrial organization, and domestic implementation arrangements, are treated as analytically relevant conditions for why their participation pathways differed.

The analysis covers the period from 2000 to 2025, capturing China’s Gavi-supported Hepatitis B Immunization Project, India’s transition from Gavi-supported programming to self-financing and renewed partnership arrangements, and the COVID-19 period, when both countries intensified engagement with Gavi and COVAX. The study aims to identify differentiated participation pathways within the same partnership framework and assess their implications for two outcome domains: domestic immunization-system strengthening and participation in global vaccine procurement.

### 2.2 Data collection

The study draws on four categories of sources. First, institutional documents and databases were collected from Gavi, WHO, UNICEF, national health authorities, and related public agencies. These materials included Gavi country and donor profiles, strategy and transition documents, media releases, the WHO Global Vaccine Market Report 2024, the WHO vaccine prequalification database, UNICEF Supply Division materials, India’s Ministry of Health and Family Welfare documents, Chinese government statements on COVAX, and China CDC releases.

Second, corporate disclosures and partnership statements were collected from vaccine manufacturers and partner organizations involved in Gavi- or COVAX-related supply, manufacturing, technology transfer, and pricing arrangements. Third, peer-reviewed studies were reviewed to provide contextual and analytical support on Gavi-supported immunization, vaccine market shaping, WHO prequalification, technology transfer, and emerging economies in global health governance. Fourth, 12 semi-structured interviews were conducted between 2019 and 2025 with vaccine industry executives, global health experts, and respondents affiliated with international philanthropic organizations. Interviewees were anonymized as industry respondents (IR), global health experts (GE), and philanthropic organization respondents (PR). Interview data were used to clarify mechanisms and triangulate documentary findings rather than as stand-alone evidence.

Materials were retained when they helped trace three elements of the analysis: role evolution within Gavi, including recipient, transition, self-financing, donor-linked, or supplier roles; mechanisms of participation, including state coordination, WHO prequalification, UNICEF–Gavi procurement, manufacturing, supply agreements, and technology transfer; and the implications of these pathways for domestic immunization strengthening and global vaccine procurement.

### 2.3 Data processing and analysis

Data processing involved three steps. First, the retained materials were cleaned and organized. Duplicate records, overlapping versions of the same institutional document, and sources without substantive information on case development were removed. The remaining materials were classified by country, source type, year, and analytical function, allowing the evidence to be organized for both chronological reconstruction and cross-case comparison.

Second, key information was extracted into a country-level chronological matrix. For each country, the matrix recorded changes in its role within Gavi, including recipient status, transition, self-financing, donor-linked engagement, and supplier participation. It also recorded the main mechanisms through which participation was organized, including state coordination, Gavi funding, co-financing arrangements, WHO prequalification, UNICEF–Gavi procurement, supply agreements, technology transfer, COVAX engagement, and domestic immunization-system interventions. Each event was then linked to one or both outcome domains: domestic immunization-system strengthening and global vaccine procurement participation.

Third, the materials were manually coded along four dimensions: role evolution, participation mechanism, pathway orientation, and outcome mapping. Role evolution captured how each country’s position within Gavi changed over time. Participation mechanism identified the institutional or market channels through which engagement occurred, such as program cooperation, state coordination, firm-level procurement integration, regulatory entry, or technology-transfer arrangements. Pathway orientation was used to distinguish state-coordinated participation from market-integrated participation. Outcome mapping assessed how each pathway was associated with domestic immunization strengthening and global vaccine procurement participation.

### 2.4 Analytical strategy and triangulation

The analysis combined process tracing and structured cross-case comparison. Process tracing was used to reconstruct the sequence of each country’s engagement with Gavi and to identify key turning points in role evolution, participation mechanisms, and outcome formation. Cross-case comparison was then used to identify differences in participation pathways under broadly similar structural conditions. Accordingly, the analysis does not claim to identify causal effects in a strict counterfactual sense. Instead, it uses process tracing and cross-case comparison to identify plausible pathway-specific associations between modes of Gavi engagement, domestic immunization-system strengthening, and participation in global vaccine procurement.

**Figure 1:**
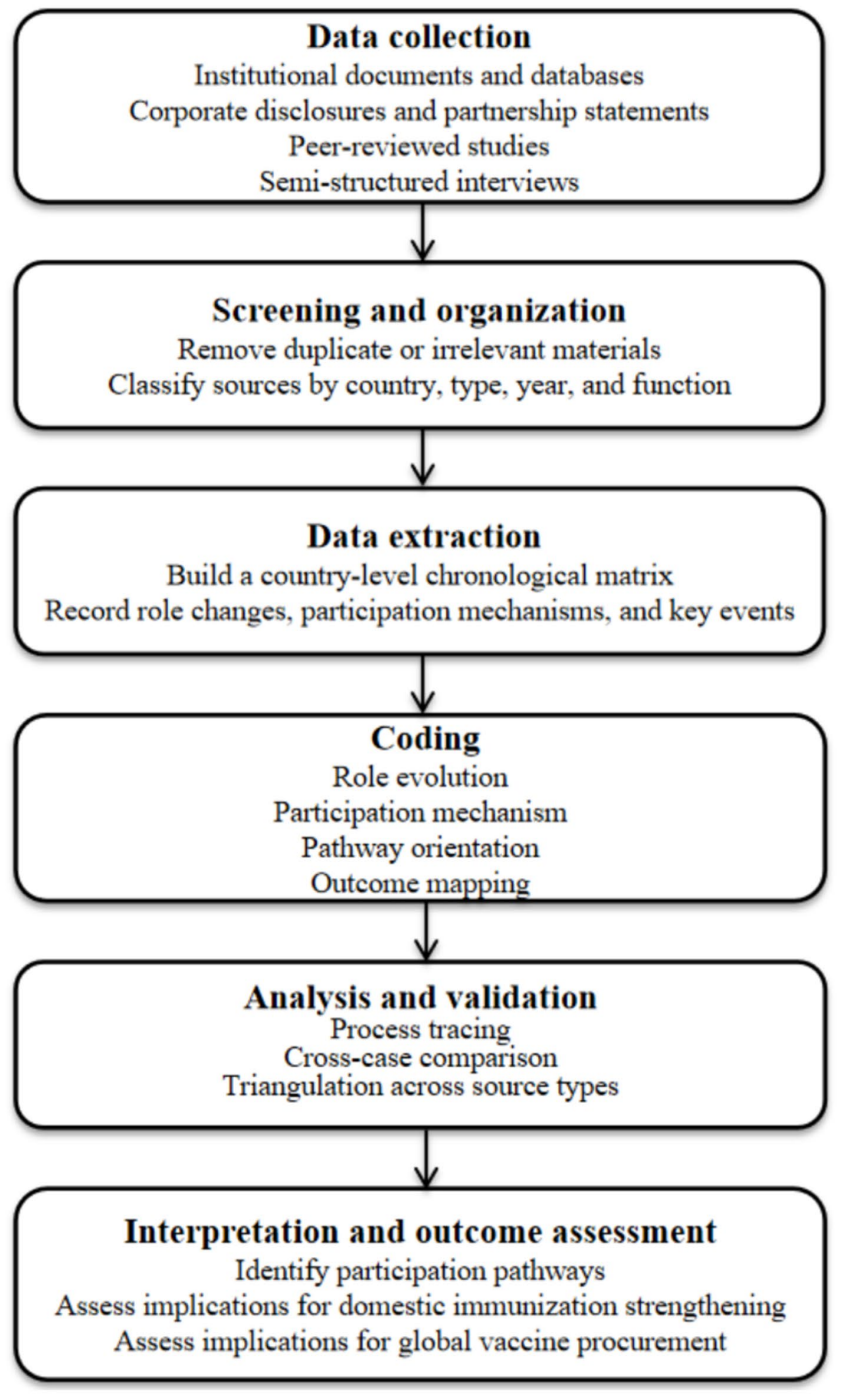
Data collection and analysis procedure. Source: Made by the author.

Triangulation was applied across institutional documents, corporate materials, peer-reviewed studies, public databases, and interview data. Claims were retained when they were supported by documentary evidence or when interview findings were consistent with multiple documentary or contextual sources. When interview evidence provided interpretation of mechanisms, it was used to clarify causal processes rather than to establish factual claims on its own. This procedure was intended to reduce reliance on any single source type and to improve the transparency and auditability of the qualitative analysis. The data collection and analysis procedure is summarized in Figure 1.

## 3 Results: divergent modes of participation

This section reports the main empirical findings of the comparison and identifies two distinct participation pathways in Gavi. China’s pathway was characterized by government-led role adjustment, fiscal commitment, and selective corporate participation. India’s pathway, by contrast, combined a continuing state role with sustained manufacturer integration into pooled procurement. The results are presented in two stages: first, China’s pathway is reconstructed from recipient-phase program cooperation to donor-oriented and policy-mediated engagement; second, India’s pathway is examined through the coexistence of continued program support, emerging contributor aspirations, and firm-led procurement embeddedness.

### 3.1 China: a state-anchored mode of engagement

China’s engagement with Gavi exhibits a clearly state-anchored pattern. Empirical evidence indicates that participation has been structured primarily through governmental policy commitments, diplomatic coordination, and fiscal arrangements, with corporate actors operating largely within a nationally defined strategic framework.

#### 3.1.1 From recipient to donor: state-led institutional repositioning

China’s initial engagement with Gavi occurred through programmatic collaboration during its period as a recipient country. In 2002, the Ministry of Health launched the China–Gavi Hepatitis B Immunization Project, with combined funding of approximately US$76 million. Over the following 8 years, the project contributed to an estimated 85% hepatitis B vaccination coverage rate and 70% timely birth-dose coverage, and was associated with the prevention of approximately 3.8 million infections and 680,000 premature deaths (16). During this phase, Gavi support functioned as complementary system strengthening, particularly in delivery capacity and immunization coverage expansion.

After this recipient phase, China’s engagement shifted toward donor-oriented participation. A visible institutional marker appeared in 2015, when China pledged US$5 million at Gavi’s Berlin Pledging Conference in support of Gavi’s 2016–2020 strategic period (17). According to Gavi’s donor profile, this US$5 million contribution marked China’s first formal donor commitment to the Alliance. Interview evidence suggests that this pledge was politically significant but not yet embedded within a sustained multi-cycle donor commitment framework (PR1, 2019).

China’s state-level engagement deepened further during the COVID-19 period. In June 2020, China pledged US$20 million to support Gavi’s 2021–2025 strategic period (17). In August 2021, China signed a US$100 million contribution agreement to support vaccine distribution to developing countries through the COVAX facility (8). Taken together, these actions show that China’s expanded participation in Gavi-related mechanisms occurred primarily through state commitments, multilateral positioning, and public financing decisions.

Three empirical features stand out from this trajectory. First, China’s engagement moved from program implementation to donor participation without sustained integration into routine pooled procurement. Second, major shifts in participation were tied to high-level policy decisions rather than to firm-led market expansion. Third, the strongest evidence of deeper engagement appeared during moments of multilateral health diplomacy, especially during the COVID-19 period. Overall, the results indicate that China’s role transition within the Gavi framework was mediated mainly through government-led institutional repositioning.

#### 3.1.2 Corporate participation under government coordination

Chinese vaccine manufacturers’ participation in Gavi-related mechanisms remained constrained along two interrelated dimensions: a limited number of WHO prequalification (PQ) products and a relatively modest scale of actual vaccine supply within Gavi-supported procurement channels.

First, in terms of regulatory entry, the number of Chinese vaccines obtaining WHO prequalification remained limited for an extended period. A notable early case emerged in 2013, when the Japanese encephalitis vaccine SA 14–14-2, produced by the Chengdu Institute of Biological Products under China National Biotec Group, obtained WHO prequalification. This was the first Chinese-made vaccine to receive WHO prequalification and enabled Gavi-supported use of the product in countries such as Lao PDR following Gavi’s approval of Japanese encephalitis vaccine support (18). Subsequent progress, however, was incremental rather than transformative. As shown in Table 1, in the years preceding the COVID-19 pandemic, only three additional Chinese vaccines obtained WHO prequalification: Hualan’s seasonal influenza vaccine in 2015, Sinopharm’s poliomyelitis vaccine in 2017, and Sinovac’s HEALIVE in 2017.

**Table 1:** Chinese vaccine products obtaining WHO PQ or WHO EUL, 2013–2022.

Period	Date	Vaccine name	Manufacturer	Status (PQ/EUL)
Before the COVID-19 pandemic	Oct 2013	Japanese Encephalitis Vaccine Live (SA14-14-2)	Chengdu Institute of Biological Products	PQ
Before the COVID-19 pandemic	June 2015	Seasonal Influenza Vaccine	Hualan Biological Engineering	PQ
Before the COVID-19 pandemic	Dec 2017	Poliomyelitis Vaccine (live, oral attenuated, human Diploid Cell)	Beijing Bio-Institute of Biological Products (Sinopharm)	PQ
Before the COVID-19 pandemic	Dec 2017	HEALIVE	Sinovac Biotech	PQ
During the COVID-19 pandemic	May 2021	SARS-CoV-2 Vaccine (Vero Cell)	Beijing Bio-Institute of Biological Products (Sinopharm)	EUL
During the COVID-19 pandemic	Jun 2021	COVID-19 Vaccine (Vero Cell)	Sinovac Biotech	EUL
During the COVID-19 pandemic	Oct 2021	Bivalent HPV Vaccine (Cecolin®)	Xiamen Innovax Biotech	PQ
During the COVID-19 pandemic	May 2022	Ad5-nCoV/Convidecia	CanSino Biologics	EUL
During the COVID-19 pandemic	Jun 2022	Poliomyelitis Vaccine (Vero Cell)	Sinovac Biotech	PQ
During the COVID-19 pandemic	Nov 2022	Varicella Vaccine (Live)	Sinovac (Dalian) Biotech	PQ

Source: WHO. Prequalified Vaccines. Available online at: https://extranet.who.int/prequal/vaccines/prequalified-vaccines (accessed on 5 February 2025).

The pandemic period marked a visible acceleration, with a denser cluster of WHO Emergency Use Listing (EUL) and PQ approvals. These included Sinopharm’s SARS-CoV-2 vaccine in May 2021, Sinovac’s COVID-19 vaccine in June 2021, Xiamen Innovax’s bivalent HPV vaccine in October 2021, CanSino’s Convidecia in May 2022, Sinovac’s Sabin-strain inactivated poliomyelitis vaccine in June 2022, and Sinovac (Dalian)‘s varicella vaccine in November 2022. Despite this acceleration, progress continued to take the form of phase-specific breakthroughs rather than large-scale and sustained PQ accumulation.

Second, in terms of actual procurement, Chinese manufacturers’ presence in Gavi-supported supply channels was limited and concentrated in a small number of identifiable episodes. An early case was Lao PDR’s 2015 Japanese encephalitis campaign, in which Gavi support enabled the introduction of a Chinese-manufactured vaccine to more than 1.5 million children (19). During the COVID-19 period, Chinese firms’ participation became more visible through the COVAX Facility. In July 2021, Gavi signed advance purchase agreements with Sinopharm and Sinovac covering an initial 110 million doses, including 60 million from Sinopharm and 50 million from Sinovac, with options for substantial additional purchases (20, 21). Chinese vaccines were subsequently delivered to recipient countries through COVAX. For instance, by the end of 2021 Pakistan had received 8,845,200 Sinopharm doses through COVAX (22). Regional data from Africa further indicate the scale of this presence. As of 4 September 2022, a total of 706 million COVID-19 vaccine doses had been delivered in the African Region, of which 66.6% were supplied through the COVAX Facility. Within this delivery structure, Sinopharm accounted for 14.2% and Sinovac for 7.8% of available vaccine doses (23).

Taken together, these cases indicate that Chinese firms did enter Gavi-supported procurement and delivery channels. However, such participation was concentrated primarily in specific programs and the pandemic period, rather than evolving into a stable presence in routine pooled procurement.

### 3.2 India: a market-embedded mode of engagement

In contrast to China’s state-coordinated pathway, India’s engagement with Gavi combined a continuing governmental role with sustained manufacturer participation in pooled procurement. India remained linked to Gavi not only through programmatic and financing arrangements, but also through the long-term presence of Indian vaccine producers in multilateral supply channels. This combination gave India a more market-integrated pattern of participation than the one observed in China.

#### 3.2.1 A hybrid state role

At the governmental level, India’s role within Gavi has evolved from that of a traditional recipient to a more hybrid position combining recipient and emerging contributor functions. Since the early 2000s, Gavi has provided sustained financial and technical support to India. According to Gavi, cumulative disbursements to India reached approximately US$1.7 billion by 2023, supporting the introduction and scale-up of hepatitis B, pentavalent, inactivated polio, rotavirus, and measles-rubella vaccines, while also supporting the introduction of HPV and typhoid conjugate vaccines (24). A pivotal shift occurred in 2017, when India initiated its graduation process and was expected to transition toward full self-financing of its vaccine programs by 2021 (25). This transition, however, did not signify withdrawal from Gavi’s financing framework. Rather, it marked a new phase in which India continued to receive targeted programmatic support while beginning to participate in Gavi replenishment discussions as an emerging contributor.

**Figure 2:**
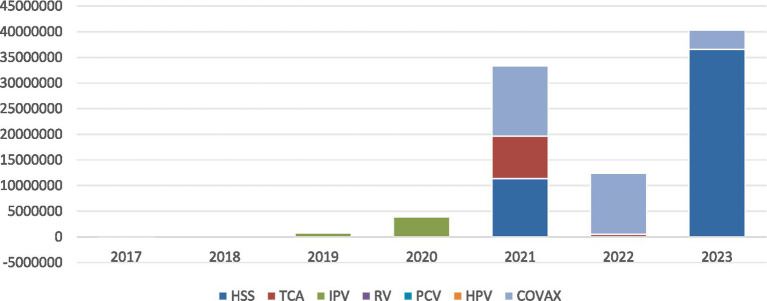
Gavi financial support to India by program area, 2017–2023 (US$). HSS, Health System Strengthening; TCA, Targeted Country Assistance; IPV, Inactivated Polio Vaccine; RV, Rotavirus Vaccine; PCV, Pneumococcal Conjugate Vaccine; HPV, Human Papillomavirus Vaccine; COVAX, COVID-19 Vaccines Global Access Facility. Source: Gavi, the Vaccine Alliance. India Country Hub. Available online at: https://www.gavi.org/programmes-impact/country-hub/south-east-asia/india (accessed on 1 February 2026).

**Figure 3:**
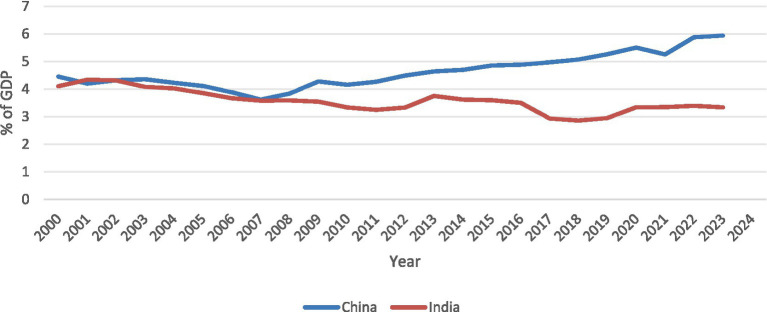
Current health expenditure (% of GDP) in China and India. Source: World Bank. Current Health Expenditure (% of GDP). Available online at: https://data.worldbank.org/indicator/SH.XPD.CHEX.GD.ZS (accessed on 11 February 2026).

On the one hand, India has continued to receive Gavi funding. Between 2017 and 2023, Gavi disbursed approximately US$ 100 million to India, as shown in Figure 2 (26). Support increased markedly following the outbreak of COVID-19, which severely disrupted routine immunization services nationwide. This pattern should be understood in the context of India’s relatively constrained public health investment. As shown in Figure 3, India’s health expenditure as a share of GDP remained below that of China, indicating a more limited domestic fiscal basis for sustaining broad-based immunization and health system development. Against this background, external support remained relevant, particularly during periods of system stress. In 2021 alone, Gavi funding to India exceeded US$ 33 million. Of this amount, more than US$ 19.6 million was allocated to Health System Strengthening (HSS) and Targeted Country Assistance (TCA), while approximately US$ 13.7 million was provided through COVAX mechanisms. This surge indicates that, despite India’s formal classification in 2022 as a fully self-financing country, critical components of its immunization system continued to rely on Gavi’s structured support for system stabilization and recovery (27).

On the other hand, India was also increasingly presented as a prospective contributor. Gavi’s donor profile records that India became the first implementing country donor in 2014, committing US$4 million over 2013–2016, US$8 million for 2018–2022, and US$15 million for Gavi’s 2021–2025 program (28). More recent reporting also suggested that the Indian government was considering a pledge of approximately US$ 20 million in response to Gavi’s emerging financing gap (29). Industry advocacy played a visible role in this development. In April 2025, the Indian Vaccine Manufacturers Association (IVMA) urged the government to commit US$ 260 million annually over 5 years (US$ 1.3 billion in total), highlighting Gavi’s importance in sustaining large-scale, affordable vaccine exports by Indian firms (29). These developments indicate that India’s gradual shift toward contributor status has been influenced not only by diplomatic repositioning but also by firm-level incentives tied to continued access to Gavi-supported procurement markets.

Overall, India’s governmental role within Gavi was not defined by a clean transition from recipient to contributor, but by the coexistence of multiple institutional roles over time. The evidence shows that Gavi’s targeted support continued after transition, even as India acquired self-financing status and developed a modest donor profile. This amounted to a hybrid state role in which program partnership, fiscal transition, and emerging contributor aspirations overlapped within the same framework.

#### 3.2.2 Firm-level participation in India

India’s firm-level participation in Gavi-supported procurement was more sustained and more structurally significant than that observed in China. The global vaccine market itself was highly concentrated: in 2023, the top 10 manufacturers accounted for approximately 73% of total doses supplied and 85% of total market value (7). Within this structure, supply was dominated by a relatively small group of producers, including Pfizer, Serum Institute of India (SII), GSK, Sanofi, Merck/MSD, and Bharat Biotech. Among them, SII was the largest supplier by volume, accounting for roughly 22% of the global vaccine market volume (7). India’s role in this system was correspondingly prominent. Approximately 60% of vaccines procured through Gavi were manufactured in India, indicating that the country had become a major production base within the Gavi–UNICEF pooled procurement system (25).

A central condition for this participation was regulatory eligibility. Access to Gavi-supported procurement was mediated primarily through WHO PQ, which functions as the main regulatory threshold for vaccines supplied through UN and Gavi-funded channels. Under this arrangement, only products meeting WHO PQ standards can enter UNICEF–Gavi pooled purchasing mechanisms. India’s national regulatory authority, the Central Drugs Standard Control Organization (CDSCO), was recognized within the WHO PQ system, and multiple Indian manufacturers obtained PQ approval across a broad range of vaccine categories (30). These manufacturers included SII, Bharat Biotech, Biological E, Panacea Biotec, Zydus Lifesciences, and Haffkine, showing that participation was not limited to a single firm but reflected a wider industrial and regulatory base. The scale of this integration is illustrated by SII’s PQ portfolio. As shown in Table 2, SII had 53 vaccine entries under WHO PQ, covering BCG, measles-containing vaccines, pentavalent and hexavalent vaccines, pneumococcal conjugate vaccines, meningococcal vaccines, influenza vaccines, malaria vaccines, COVID-19 vaccines, and inactivated polio vaccine. This portfolio indicates that Indian manufacturers were not confined to a narrow set of basic products, but were able to supply both routine childhood vaccines and more complex platforms.

**Table 2:** WHO-prequalified vaccines produced by Serum Institute of India.

Vaccine category	Number of PQ entries
BCG	1
COVID-19	1
Malaria	1
Diphtheria–Tetanus (DT, pediatric)	3
Diphtheria–Tetanus (reduced antigen content, adults/adolescents)	3
Diphtheria–Tetanus–Pertussis (whole-cell DTP)	3
Pentavalent (DTP–Hib)	1
Pentavalent (DTP–HepB–Hib)	3
Pentavalent (DTP–HepB–Hib, adsorbed)	3
Hexavalent (DTP–HepB–Hib–IPV)	2
*Haemophilus influenzae* type b (Hib)	1
Hepatitis B (adult & pediatric formulations)	4
Measles	4
Measles–Rubella (MR)	4
Measles–Mumps–Rubella (MMR)	4
Meningococcal ACYWX conjugate (MenFive™)	2
Meningococcal A conjugate (MenAfriVac®)	2
Influenza, pandemic H1N1 (NASOVAC)	2
Influenza, seasonal (trivalent)	1
Pneumococcal conjugate (PCV, PNEUMOSIL®)	3
Polio vaccine – Inactivated (IPV)	2
Total	53

Source: WHO. Prequalified Vaccines. Available online at: https://extranet.who.int/prequal/vaccines/prequalified-vaccines (accessed on 5 February 2025).

Technology-transfer partnerships further expanded the range and scale of Indian firm participation. Although Indian manufacturers were less prominent in the original development of some novel vaccine platforms, they were able to enter Gavi-supported supply channels through licensing and manufacturing agreements with multinational pharmaceutical companies (31). During the COVID-19 pandemic, AstraZeneca entered into a licensing arrangement with the Serum Institute of India (SII) to supply up to 1 billion doses for low- and middle-income countries (32). Under this arrangement, SII became a contracted supplier to COVAX and produced the AstraZeneca–Oxford vaccine (COVISHIELD) for 64 lower-income economies participating in the Gavi COVAX Advance Market Commitments (AMCs), while also serving domestic demand in India (33). By March 2021, COVAX had received 28 million doses of COVISHIELD. A similar pattern appeared in malaria vaccine production. In 2025, Bharat Biotech and GSK announced commitments linked to Gavi’s 2026–2030 replenishment, including a planned RTS, S price reduction, technology transfer, and capacity expansion at Bharat Biotech (34).

Firm participation was also supported by cost and scale advantages. Compared with producers in high-income settings, Indian manufacturers operated with lower labor, facility, depreciation, and clinical trial costs, which supported large-volume production at comparatively low unit prices (GE1, 2022) (35, 36). Leading firms such as the Serum Institute of India (SII) have expanded capacity and optimized production processes while meeting WHO PQ standards, thereby “sustaining competitive pricing within multilateral procurement markets” (IR1, 2020). SII’s pneumococcal conjugate vaccine (Pneumosil) is widely cited as one of the most price-competitive PCVs globally; its agreement with UNICEF reduced the per-dose price to approximately US$ 2, a benchmark highlighted by UNICEF as a case of improved affordability for low-income countries (37). This pricing position is not subsidy-driven but scale-driven. Current annual production capacity is estimated at approximately 3 billion doses for SII, 4 billion for Bharat Biotech, and 1 billion for Panacea Biotec (38). Through sustained high-volume output that spreads fixed costs across large production runs, Indian manufacturers have consolidated a stable “low-cost, high-volume” supply model, enabling their continued embeddedness in the global vaccine procurement market.

This pattern was reinforced by the structure of Gavi-supported procurement itself. Through pooled procurement and AMCs, demand from multiple low- and middle-income countries was aggregated into larger and more predictable purchasing volumes (PR2, 2023). UNICEF, as Gavi’s primary procurement agency, aggregates country demand, negotiates prices, and establishes long-term framework agreements that secure volume commitments and enable scale-based price reductions (39). Gavi’s AMCs mechanism further reduces market uncertainty by guaranteeing minimum purchase volumes and signaling future demand, thereby mitigating financial risk associated with capacity expansion and capital investment (40). Within this procurement environment, Indian manufacturers were well positioned because they combined WHO-prequalified products, large-scale output, and competitive pricing across multiple vaccine categories. The results therefore show that India’s firm-led participation in Gavi was not limited to individual contracts, but was supported by a broader alignment between domestic manufacturing capabilities and the institutional design of multilateral vaccine procurement.

### 3.3 Cross-case summary of participation patterns

The comparison identifies two distinct participation pathways within the Gavi framework. In China, engagement was shaped primarily by state coordination, programmatic cooperation, and donor-oriented fiscal commitments, while corporate participation remained selective and was activated mainly through regulatory openings or crisis-specific opportunities. In India, by contrast, participation combined a continuing governmental role with sustained firm-level integration into pooled procurement. Government engagement evolved through transition, self-financing, and selective donor signaling, while manufacturers remained deeply involved in vaccine supply through WHO-prequalified portfolios, licensing arrangements, and large-scale production.

These differences were visible across several dimensions. First, the main organizational actor differed. In China, the state remained the principal actor linking domestic immunization policy with Gavi-related engagement. In India, state institutions continued to matter, but long-term manufacturer participation was more central to the country’s position within the partnership. Second, the dominant mode of participation also differed. China’s engagement was expressed mainly through projects, policy commitments, and selective multilateral coordination, whereas India’s engagement was more strongly expressed through routine procurement participation and supply continuity. Third, the relationship between global participation and domestic immunization systems followed different patterns. In China, Gavi-related engagement was more closely aligned with domestic system development during the recipient phase and with state-led multilateral positioning thereafter. In India, by contrast, strong participation in global vaccine procurement coexisted with a more uneven pattern of domestic immunization system strengthening.

Overall, the results suggest that participation in Gavi among emerging economies does not follow a single trajectory. Instead, the comparison shows that similar countries can combine domestic capacity, state strategy, and market participation in different ways, associated with different public health and procurement-related outcomes. Table 3 summarizes the main empirical contrasts between the two cases.

**Table 3:** Comparative summary of China’s and India’s participation pathways in Gavi: key patterns and supporting indicators.

Dimension	China	India
Predominant actor configuration	Predominantly state-led, with selective policy-mediated corporate participation	Predominantly firm-led, alongside continuing governmental engagement with Gavi
Governmental role in Gavi	Recipient-phase cooperation; later donor-oriented and policy-coordinated engagement	Long-standing recipient/program partner; transition to self-financing; emerging donor role
Key state-level financial commitments	China-Gavi HepB project: ~US$76 million; donor pledges of US$5 million (2015), US$20 million (2020), and US$100 million for COVAX (2022)	Cumulative Gavi disbursements to India: ~US$1.7 billion by 2023; ~US$100 million disbursed during 2017–2023; donor commitments of US$4 million (2013–2016) and US$8 million (2018–2022)
Corporate participation pattern	Selective, policy-mediated, and episodic	Sustained, firm-led, and procurement-integrated
Regulatory entry into Gavi-related supply	10 Chinese vaccine PQ/EUL entries identified for 2013–2022; visible acceleration only during the pandemic period	Broad PQ-based regulatory integration; SII alone held 53 WHO PQ entries across multiple vaccine categories
Scale of procurement participation	Lao PDR JE campaign covered >1.5 million children; 2021 COVAX agreements covered an initial 110 million doses; Pakistan received 8,845,200 Sinopharm doses by end-2021	~60% of Gavi-procured vaccines manufactured in India; SII accounted for ~22% of globally procured doses in 2023; COVAX had received 28 million doses of COVISHIELD by March 2021
Form of market integration	Entry mainly through specific programs and pandemic emergency windows	Long-term embeddedness in pooled procurement supported by WHO PQ, licensing partnerships, and scale production
Overall participation pathway	State-anchored and policy-mediated, with selective market entry	Market-embedded and firm-integrated, alongside a hybrid governmental role

Source: Author’s summary based on findings presented in Section 3.

## 4 Discussion

This study examined how different patterns of engagement with Gavi are associated with domestic immunization system development and positions within global vaccine procurement. By comparing China and India over the period 2000–2025, the analysis shows that participation in Gavi is not organized through a single pathway among emerging economies. Instead, countries with broadly comparable production capacity and long-standing links to Gavi may combine state coordination, programmatic support, and market participation in markedly different ways. This matters because these pathways are associated with different public health consequences: some are more closely linked to domestic immunization system strengthening, whereas others are more strongly associated with sustained participation in multilateral vaccine supply.

### 4.1 China: state-coordinated engagement and domestic immunization system strengthening

The Chinese case illustrates a state-coordinated mode of engagement in which participation in Gavi was shaped by China’s centralized public-health governance structure. Rather than relying primarily on autonomous firm-level participation in multilateral procurement markets, China’s engagement was mediated through state institutions, national technical agencies, and a vertically organized public-health delivery system. This mode of participation was associated with closer alignment between external partnership resources and domestic immunization priorities. At the same time, it limited the extent to which Chinese vaccine manufacturers developed durable, recurrent participation in Gavi-related pooled procurement channels.

**Figure 4:**
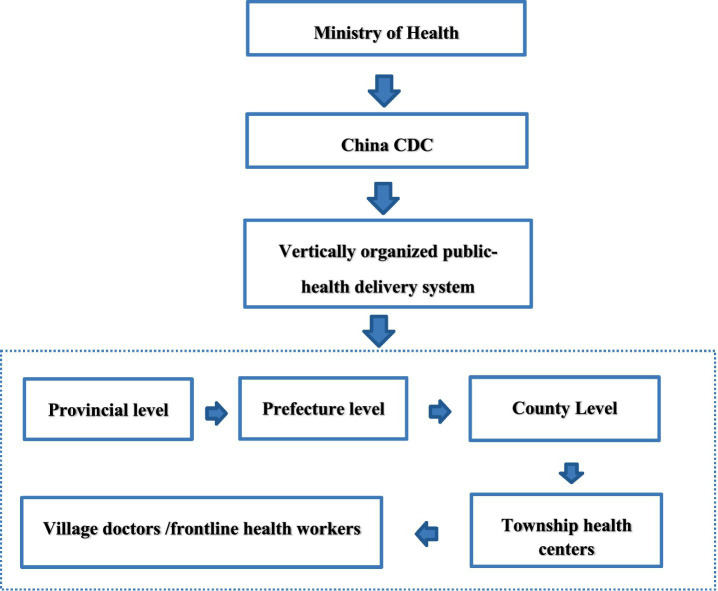
China’s vertically organized public health system. Source: Author’s own elaboration based on Cui et al. (43).

This state-coordinated pattern was rooted in China’s public-health governance architecture during the earlier Gavi recipient phase. As shown in Figure 4, the Ministry of Health functioned as the main national policy and administrative interface for immunization-related cooperation, while China CDC provided technical support for disease control, immunization guidance, training, monitoring, and evaluation. Implementation then proceeded through a vertically organized public-health delivery system linking provincial, prefecture, county, township, and village-level health authorities and frontline health workers (41). This appeared to support alignment between external support and national immunization priorities and was associated with implementation through an existing delivery system.

The Gavi-supported hepatitis B immunization project illustrates how external resources were incorporated into domestic system-building through this institutional structure. China’s participation was not limited to receiving external financial assistance. The Chinese government financed 50% of project costs, a dedicated project office was established in China to support program management, training, and coordination, and project funding was used for the local procurement of domestically produced hepatitis B vaccines and auto-disable syringes (41). These arrangements embedded Gavi support within China’s immunization system rather than leaving it as a stand-alone aid intervention.

This institutional embedding was closely linked to implementation patterns. Gavi resources supported not only vaccine procurement, but also service delivery, workforce development, project monitoring, and injection safety (42). Project savings were redirected to demonstration activities aimed at improving timely birth-dose coverage, training Expanded Program on Immunization (EPI) staff, and monitoring project impact. These investments were accompanied by measurable improvements in local capacity: county-level human resources engaged in hepatitis B vaccination increased from an average of 29 per county in 2002 to 66 in 2009; all Gavi-funded project counties used auto-disable syringes for hepatitis B vaccination and other vaccines; and surveyed infant hepatitis B vaccine coverage increased from 71% in 2002 to 93% in 2009 (42).

A CDC report further suggests that improvements in timely birth-dose coverage were associated with systematic support measures implemented through China’s vertically organized public health system, including expanded vaccine availability in hospitals and township health facilities, stronger coordination between delivery services and vaccination services, intensified training, supervision and monitoring at county, township and village levels, and subsidies for village doctors to provide vaccines (43).

Interview evidence is consistent with this interpretation. One interviewee noted that Gavi-related engagement in China was often understood as “contributing to wider public health system development” rather than “narrowly to vaccine supply” (GE2, 2019). Other interviews further suggest that centralized coordination reduced the institutional burden on multilateral partners. Global health initiatives involving China were typically reviewed and coordinated at the central government level, which “reduced the need for multilateral organizations to navigate multiple domestic stakeholders” (GE3, 2025). Similarly, this coordination structure helped “lower transaction costs” and “mitigate institutional friction between multilateral programs and domestic governance processes” (PR2, 2023). In this sense, state coordination appeared to support policy alignment between China’s domestic governance architecture and multilateral health mechanisms.

However, the same organizational logic also constrained China’s long-term procurement embeddedness. Because engagement with Gavi-related mechanisms was largely mediated by the state, Chinese manufacturers tended to treat participation less as an autonomous commercial strategy than as part of a government-coordinated policy process. As one industry respondent explained, firms generally “looked to the state to manage overall engagement with Gavi,” rather than developing the capacity to “independently navigate a complex multilateral procurement system” (IR1, 2020). This reliance on state coordination may have limited the incentives and experience needed for firms to engage recurrently with Gavi’s procurement rules, product requirements, tender cycles, and supplier relationships. This matters because Gavi procurement depends on recurrent supplier engagement, not occasional supply: Gavi uses demand forecasting and supply planning to provide market visibility, while UNICEF Supply Division procures the majority of Gavi-funded vaccines and works with manufacturers to secure reliable, affordable, and quality-assured supply (44).

As a result, Chinese manufacturers’ participation remained selective and episodic, often occurring when government coordination, policy priorities, or exceptional international circumstances created an opening. The COVAX episode, discussed above, reinforces this pattern. Although Sinopharm and Sinovac became more visible through COVAX supply agreements, Gavi framed these July 2021 advance purchase agreements as measures to provide immediate COVAX supply and address critical supply gaps (21). This episode therefore represented an important but exceptional expansion of Chinese supply, rather than durable firm-level integration into Gavi’s routine pooled procurement channels.

The Chinese pathway therefore reveals an important trade-off. State-coordinated engagement was associated with the alignment of external partnership resources with domestic immunization priorities and their incorporation into local implementation capacity. At the same time, this approach was less effective in fostering recurrent, autonomous firm-level participation in Gavi’s routine procurement channels. China therefore did not simply move from recipient status to a conventional donor or supplier role. Instead, Gavi participation appeared to serve primarily as a channel for domestic system consolidation, while also allowing China to expand its role in global health governance in a gradual and selective manner.

### 4.2 India: market-integrated participation and global vaccine procurement

India’s engagement with Gavi reflects a market-integrated pathway of participation. Unlike China’s more state-coordinated trajectory, which was closely associated with domestic institutional consolidation, India’s participation was mediated primarily through firm-level integration into the UNICEF–Gavi pooled procurement system. This pathway was associated with two broader patterns. First, it coincided with Indian manufacturers’ position as globally important vaccine suppliers, especially within aid-funded and pooled procurement markets. Second, it shows that participation in a vaccine partnership does not necessarily produce uniform public health effects. External procurement influence may coexist with uneven domestic immunization-system consolidation.

As shown in Section 3.2, Indian manufacturers came to occupy a structurally significant position within the UNICEF–Gavi procurement architecture. Through WHO-prequalified products, large-scale production capacity, technology-transfer arrangements, and competitive pricing across multiple vaccine categories, they became embedded not simply as low-cost suppliers, but as recurrent participants in a procurement system designed to serve low- and lower-middle-income countries. This embeddedness is analytically important because Gavi-supported procurement does not necessarily end when grant financing declines. It may create continuity between aid-financed demand and subsequent market-based procurement.

Gavi’s transition framework allows countries graduating from Gavi support to continue procuring vaccines either through UNICEF or through national self-financing mechanisms, rather than discontinuing established supply relationships once grant financing ends (45). Empirical evidence from UNICEF market reports illustrates this continuity. In the rotavirus vaccine market, for example, UNICEF reported that, alongside Gavi-supported countries, 14 countries that had transitioned from Gavi support continued to access vaccines through UNICEF on a self-financing basis (46). For Indian manufacturers, repeated participation in UNICEF–Gavi procurement channels therefore offered more than aid-funded sales. It may have allowed firms to accumulate regulatory credibility, delivery records, and buyer relationships that could support longer-term market positioning in countries gradually moving beyond Gavi support.

Interview evidence reinforces this interpretation at the firm level. One respondent noted that large-scale UNICEF and Gavi contracts appeared to enable Indian manufacturers not only to “strengthen their position in aid-funded markets” but also to “access broader demand in recipient countries” (IR1, 2020). The same respondent noted that established supply records created “durable commercial footholds”, as recipient governments often continued sourcing from incumbent suppliers when multilateral support declined or phased out (IR1, 2020). This suggests a public procurement–market entry–path dependency mechanism: multilateral purchasing did not merely provide short-term procurement opportunities, but may have supported Indian firms’ more durable commercial presence beyond the initial aid-financed phase.

At the same time, the Indian case reveals the limits of a firm-led, market-integrated pathway. Gavi’s support to India was not confined to vaccine procurement. It was also channeled into system-oriented interventions linked to new vaccine introduction. For instance, during the IPV rollout, India received Gavi support for the 2015–2018 period, with an estimated program cost of US$160 million, and implementation was gradually expanded from selected states to nationwide coverage (47). In parallel, Gavi provided approximately US$30.6 million for IPV introduction and US$107 million for health system strengthening in 2014–2015 (48). These resources were directed toward underperforming states and districts and used to strengthen cold-chain infrastructure, vaccine logistics and information systems such as Electronic Vaccine Intelligence Network (eVIN), workforce capacity, monitoring and evaluation, and demand-generation activities (48).

These interventions were not external add-ons, but were incorporated into India’s existing immunization architecture and aligned with national initiatives such as Mission Indradhanush (PR2, 2023). Their effects, however, remained uneven. This unevenness reflects a broader institutional condition: external support can facilitate vaccine introduction and system upgrading, but its long-term effect depends on domestic absorption, financing, and implementation capacity (49). In India, central authorities could set national priorities and coordinate with Gavi, but program delivery depended heavily on state and district health systems. This multilevel structure created significant variation in implementation capacity and coverage outcomes. Recent data show that India’s national full immunization coverage reached 93.5% in FY 2023–2024, but state-level performance ranged from 48.03% in Dadra and Nagar Haveli and Daman and Diu, 59.62% in Puducherry, and 62.18% in Nagaland to more than 98% in Karnataka, Uttar Pradesh, Tripura, and West Bengal (50).

The Indian experience therefore suggests a partial decoupling between supply-side industrial capacity and delivery-side public health governance. Manufacturing strength may enhance a country’s external position in global procurement, but domestic immunization performance depends on a different set of capabilities: fiscal absorption, subnational implementation, service delivery, last-mile cold-chain management, demand generation, and the ability to reach underserved populations. The COVID-19 period made this distinction especially visible, as manufacturing capacity alone was insufficient to ensure timely and equitable vaccine access within the country (51). For public health policy, the implication is clear: stronger participation in global supply arrangements should not be assumed to translate automatically into stronger immunization governance at home. In India’s case, firm-led integration into procurement was associated with clear gains in external market position and supply relevance, but these gains coexisted with a more uneven pattern of domestic health-system consolidation.

### 4.3 Limitations and future research

Several limitations should be noted. First, although the analysis draws on multiple sources—including institutional documents, public databases, corporate materials, and interviews—some aspects of bargaining, coordination, and informal influence within pooled procurement arrangements remain difficult to observe directly. Second, interview evidence was useful for interpreting institutional processes, but such accounts may still reflect the perspectives and positioning of specific respondents. Third, the two-case comparative design helped identify contrasting participation patterns in depth, yet the findings should be interpreted with caution beyond large emerging economies with relatively strong manufacturing capacity and distinctive state–market relationships. Finally, although the most-similar systems logic helps structure the comparison, China and India differ in important structural respects, including state capacity, federal or centralized implementation arrangements, public health financing, and industrial organization. The findings should therefore be interpreted as an analytical comparison of participation pathways rather than as a controlled causal test that isolates one single explanatory variable.

Further research could build on this analysis in several directions. One avenue would be to extend the comparison to a wider range of country cases, particularly those with weaker production capacity or different forms of engagement with Gavi. Another would be to incorporate longer-term indicators, such as vaccine-specific price trends, shifts in supplier concentration within pooled procurement, and the durability of immunization systems after transition or graduation. It would also be useful to distinguish more clearly between routine immunization dynamics and crisis-related participation during major health emergencies, as these settings may generate different incentives, constraints, and policy effects.

## 5 Conclusion and public health policy implications

This study suggests that emerging economies do not engage with Gavi through a single pathway. China and India illustrate two differentiated forms of participation within the same partnership framework. India followed a more market-integrated pathway: sustained participation by Indian manufacturers in UNICEF–Gavi pooled procurement was associated with India’s greater relevance in global vaccine supply and appeared to support the position of Indian firms in aid-funded and multilateral vaccine markets. China, by contrast, followed a more state-coordinated pathway. Its engagement with Gavi was more closely associated with program-based cooperation, public-sector coordination, and the incorporation of external support into China’s centrally coordinated national immunization system. This comparison suggests that participation in global vaccine partnerships is shaped not only by economic capacity, but also by the practical configuration of state authority, industrial organization, regulatory capacity, and procurement arrangements. For emerging vaccine-producing countries in the Global South, neither pathway should be treated as a universal model. The key policy question is not whether China’s or India’s experience should be replicated, but how global engagement can be sequenced and aligned with domestic public health priorities under different institutional conditions. Three implications follow.

First, governments should strengthen the delivery foundations of immunization systems before assuming that manufacturing capacity or external partnership participation is sufficient to support durable health gains. Cold-chain capacity, last-mile service delivery, health workforce training, disease surveillance, vaccine safety systems, and immunization information systems are essential for translating vaccine supply into population-level protection. This is consistent with the Immunization Agenda 2030, which emphasizes resilient immunization programs integrated with primary health care and oriented toward equity and life-course coverage (52).

Second, regulatory upgrading and firm-level internationalization should proceed together. WHO prequalification is a critical gateway to United Nations vaccine procurement because it provides assurance that vaccine products meet international standards of safety, effectiveness, and quality (53). Regulatory capacity, however, should not be treated only as an export-enabling tool. It is also a domestic public health investment. Regulatory upgrading can contribute to vaccine safety, quality assurance, pharmacovigilance, lot release, and the credibility of national immunization programs (53). Governments should therefore create enabling conditions for firm internationalization through predictable regulatory rules, technical support for WHO prequalification, international registration assistance, procurement information services, and risk-sharing mechanisms. Vaccine firms, in turn, need to develop their own product portfolios, pricing strategies, certification plans, and market relationships.

Third, industrial upgrading and technology transfer should be tied directly to public health objectives. For countries that cannot immediately enter pooled procurement at scale, selective partnerships with established manufacturers may help build production capacity, quality-management experience, and international certification readiness. However, such partnerships should not be driven only by export opportunities. They should be connected to routine immunization needs, access to priority vaccines, procurement planning, and emergency supply preparedness. For countries that already possess export-oriented vaccine industries, manufacturing capacity and international market position should not substitute for sustained investment in service delivery and equitable access. Gavi’s market-shaping work aims to improve vaccine affordability, supply sustainability, and demand predictability. These market-level gains are more likely to translate into population-level health benefits only when matched by country-level delivery capacity (54).

## Data Availability

The raw data supporting the conclusions of this article will be made available by the authors, without undue reservation.
